# On the regularization of impact without collision: the Painlevé paradox and compliance

**DOI:** 10.1098/rspa.2016.0773

**Published:** 2017-06-14

**Authors:** S. J. Hogan, K. Uldall Kristiansen

**Affiliations:** 1 Department of Engineering Mathematics, University of Bristol, Bristol BS8 1UB, UK; 2 Department of Applied Mathematics and Computer Science, Technical University of Denmark, 2800 Kongens Lyngby, Denmark

**Keywords:** Painlevé paradox, impact without collision, compliance, regularization

## Abstract

We consider the problem of a rigid body, subject to a unilateral constraint, in the presence of Coulomb friction. We regularize the problem by assuming compliance (with both stiffness and damping) at the point of contact, for a general class of normal reaction forces. Using a rigorous mathematical approach, we recover impact without collision (IWC) in both the inconsistent and the indeterminate Painlevé paradoxes, in the latter case giving an exact formula for conditions that separate IWC and lift-off. We solve the problem for arbitrary values of the compliance damping and give explicit asymptotic expressions in the limiting cases of small and large damping, all for a large class of rigid bodies.

## 1. Introduction

**Figure 1.**
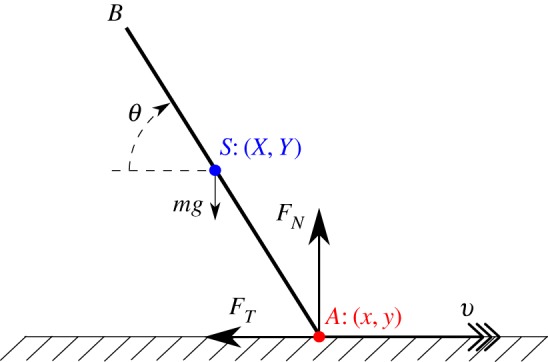
The classical Painlevé problem.

In mechanics, in problems with unilateral constraints in the presence of friction, the rigid-body assumption can result in the governing equations having multiple solutions (the *indeterminate* case) or no solutions (the *inconsistent* case). The classical example of Painlevé [1–3], consisting of a slender rod slipping^1^ along a rough surface (figure 1), is the simplest and most studied example of these phenomena, now known collectively as *Painlevé paradoxes* [5–8]. Such paradoxes can occur at physically realistic parameter values in many important engineering systems [9–15].

When a system has no *consistent* solution, it cannot remain in that state. Lecornu [16] proposed a jump in vertical velocity to escape an inconsistent, horizontal velocity, state. This jump has been called *impact without collision* (IWC) [17], *tangential impact* [18] or *dynamic jamming* [13]. Experimental evidence of IWC is given in [15]. IWC can be incorporated into the rigid-body formulation [19,20] by considering the equations of motion in terms of the normal impulse, rather than time.

Génot & Brogliato [17] considered the dynamics around a critical point, corresponding to zero vertical acceleration of the end of the rod. They proved that, when starting in a consistent state, the rod must stop slipping before reaching the critical point. In particular, paradoxical situations cannot be reached after a period of slipping.

One way to address the Painlevé paradox is to *regularize* the rigid-body formalism. Physically, this often corresponds to assuming some sort of compliance at the contact point *A*, typically thought of as a spring, with stiffness (and sometimes damping) that tends to the rigid body model in a suitable limit. Mathematically, very little rigorous work has been done on how IWC and Painlevé paradoxes can be regularized. Dupont & Yamajako [21] treated the problem as a slow–fast system, as we will do. They explored the fast time-scale dynamics, which is unstable for the Painlevé paradoxes. Song *et al.* [22] established conditions under which these dynamics can be stabilized. Le Suan An [23] considered a system with bilateral constraints and showed qualitatively the presence of a regularized IWC as a jump in vertical velocity from a compliance model with diverging stiffness. Zhao *et al.* [24] considered the example in figure 1 and regularized the equations by assuming a compliance that consisted of an *undamped* spring. They estimated, as a function of the stiffness, the orders of magnitude of the time taken in each phase of the (regularized) IWC. Another type of regularization was considered by Neimark & Smirnova [25], who assumed that the normal and tangential reactions took (different) finite times to adjust.

In this paper, we present the first rigorous analysis of the regularized rigid-body formalism, in the presence of compliance with both stiffness *and* damping. We recover IWC in both the inconsistent and the indeterminate cases, and in the latter case, we present a formula for conditions that separate IWC and lift-off. We solve the problem for arbitrary values of the compliance damping and give explicit asymptotic expressions in the limiting cases of small and large damping. Our results apply directly to a general class of rigid bodies. Our approach is similar to that used in [26,27] to understand the forward problem in piecewise smooth (PWS) systems in the presence of a twofold.

The paper is organized as follows. In §2, we introduce the problem, outline some of the main results known to date and include compliance. In §3, we give a summary of our main results, theorems 3.1 and 3.2, before presenting their derivation in §§4 and 5. We discuss our results in §6 and outline our conclusion in §7.

## 2. Classical Painlevé problem

Consider a rigid rod *AB*, slipping on a rough horizontal surface, as depicted in figure 1.

The rod has mass *m*, length 2*l*, the moment of inertia of the rod about its centre of mass *S* is given by *I* and its centre of mass coincides with its centre of gravity. The point *S* has coordinates (*X*,*Y*) relative to an inertial frame of reference (*x*,*y*) fixed in the rough surface. The rod makes an angle *θ* with respect to the horizontal, with *θ* increasing in a clockwise direction. At *A*, the rod experiences a contact force (−*F*_*T*_,*F*_*N*_), which opposes the motion. The dynamics of the rod is then governed by the following equations:

2.1 $$\begin{array}{rl} m\ddot{X} & =-F_{T},\\ m\ddot{Y} & =-mg+F_{N}\\ \;\mathrm{and}\;I\ddot{\theta } & =-l(\cos\;\theta F_{N}-\sin\;\theta F_{T}), \end{array}\}$$

where *g* is the acceleration due to gravity, plus the unilateral constraint *y*≥0.

The coordinates (*X*,*Y*) and (*x*,*y*) are related geometrically as follows:

2.2 x=X+l cos θandy=Y−l sin θ.

We now adopt the scalings $\left(X,Y)=l(\tilde{X\;}\;,\tilde{Y\;}\;),(x,y)=l(\tilde{x},\tilde{y}),(F_{T},F_{N})=mg({\tilde{F}}_{T},{\tilde{F}}_{N}\right)$, $t=1/\omega \tilde{t},\alpha =ml^{2}/I$, where *ω*^2^=*g*/*l*. For a uniform rod, $I=\frac{1}{3}ml^{2}$, and so *α*=3 in this case.

Then for general *α*, (2.1) and (2.2) can be combined to become, on dropping the tildes,

2.3 $$\begin{array}{rl} \ddot{x} & =-{\dot{\theta }}^{2}\;\cos\;\theta +\alpha \;\sin\;\theta \;\cos\;\theta F_{N}-(1+\alpha \;\sin^{2}\;\theta )F_{T},\\ \ddot{y} & =-1+{\dot{\theta }}^{2}\;\sin\;\theta +(1+\alpha \;\;\cos^{2}\;\theta )F_{N}-\alpha \;\sin\;\theta \;\cos\;\theta F_{T}\\ \;\mathrm{and}\;\ddot{\theta } & =-\alpha (\cos\;\theta F_{N}-\;\sin\;\theta F_{T}). \end{array}\}$$

To proceed, we need to determine the relationship between *F*_*N*_ and *F*_*T*_. We assume Coulomb friction between the rod and the surface. Hence, when $\dot{x}\neq 0$, we set

2.4 $$F_{T}=\mu \;\text{sign}(\dot{x})F_{N},$$

where *μ* is the coefficient of friction. By substituting (2.4) into (2.3), we obtain two sets of governing equations for the motion, depending on the sign of $\dot{x}$, as follows:

2.5 $$\begin{array}{rl} \dot{x} & =v,\;\dot{v}=a(\theta ,\phi )+q_{\pm }(\theta )F_{N},\;\dot{y}=w,\\ \dot{w} & =b(\theta ,\phi )+p_{\pm }(\theta )F_{N},\;\dot{\theta }=\phi \;\text{and}\;\dot{\phi }=c_{\pm }(\theta )F_{N}, \end{array}\}$$

where the variables *v*,*w*,*ϕ* denote velocities in the *x*,*y*,*θ* directions, respectively, and

2.6 $$\begin{array}{rl} a(\theta ,\phi ) & =-\phi^{2}\;\cos\;\theta ,\;q_{\pm }(\theta )=\alpha \;\sin\;\theta \;\cos\;\theta \mp \mu (1+\alpha \;\sin^{2}\;\theta ),\\ b(\theta ,\phi ) & =-1+\phi^{2}\;\sin\;\theta ,\;p_{\pm }(\theta )=1+\alpha \;\cos^{2}\;\theta \mp \mu \alpha \;\sin\;\theta \;\cos\;\theta \\ \;\mathrm{and}\;c_{\pm }(\theta ) & =-\alpha (\cos\;\theta \mp \mu \;\sin\;\theta ) \end{array}\}$$

for the configuration in figure 1. The suffices ± correspond to $\dot{x}=v≷0$, respectively.

Suppose *F*_*N*_ is known. Then system (2.5) is a Filippov system [4]. Hence, we obtain a well-defined forward flow when $\dot{x}=v=0$ and

2.7 $$a(\theta ,\phi )+q_{+}(\theta )F_{N}<0<a(\theta ,\phi )+q_{-}(\theta )F_{N},$$

where $\dot{v}$ in (2.5)_±_ for v≷0 both oppose *v*=0, by using the Filippov vector-field [4]. Simple computations give the following:

**Proposition 2.1.**

*The Filippov vector-field, within the subset of the switching manifold* $\Sigma :\dot{x}=v=0$ *where* (*2.7*) *holds, is given by*

2.8 $$\begin{array}{rl} \dot{y} & =w,\;\dot{w}=b(\theta ,\phi )+S_{w}(\theta )F_{N},\\ \dot{\theta } & =\phi \;\mathrm{and}\;\dot{\phi }=S_{\phi }(\theta )F_{N}, \end{array}\}$$

*where*

2.9 $$\begin{array}{rl} S_{w}(\theta ) & =\frac{q_{-}(\theta )}{q_{-}(\theta )-q_{+}(\theta )}p_{+}(\theta )-\frac{q_{+}(\theta )}{q_{-}(\theta )-q_{+}(\theta )}p_{-}(\theta )=\frac{1+\alpha }{1+\alpha \;\sin^{2}\;\theta }\\ \;\mathrm{and}\;S_{\phi }(\theta ) & =\frac{q_{-}(\theta )}{q_{-}(\theta )-q_{+}(\theta )}c_{+}(\theta )-\frac{q_{+}(\theta )}{q_{-}(\theta )-q_{+}(\theta )}c_{-}(\theta )=-\frac{\alpha \;\cos\;\theta }{1+\alpha \;\sin^{2}\;\theta }. \end{array}\}$$

**Remark 2.2.**

Our results hold for mechanical systems with different *q*_±_, *p*_±_ and *c*_±_ in (2.6) and even dependency on several angles $\theta \in T^{d}$, e.g. the two-link mechanism of Zhao *et al.* [15]. As expected, *S*_*w*_ and *S*_*ϕ*_ in (2.9) are independent of *μ*, even for general *q*_±_, *p*_±_ and *c*_±_.

To solve (2.5) and (2.8), we need to determine *F*_*N*_. The constraint-based method leads to the Painlevé paradox. The compliance-based method is the subject of this paper.

### (a) Constraint-based method

In order that the constraint *y*=0 be maintained, $\ddot{y}(=\dot{w})$ and *F*_*N*_ form a complementarity pair given by

2.10 $$\dot{w}\geq 0,\;F_{N}\geq 0,\;F_{N}\cdot \dot{w}=0.$$

Note that *F*_*N*_≥0 since the rough surface can only push, not pull, the rod. Then for general motion of the rod, *F*_*N*_ and *y* satisfy the complementarity conditions

2.11 $$0\leq F_{N}⊥y\geq 0.$$

In other words, at most one of *F*_*N*_ and *y* can be positive.

For the system shown in figure 1, the Painlevé paradox occurs when *v*>0 and *θ*∈(0,*π*/2), provided *p*_+_(*θ*)<0, as follows. From the fourth equation in (2.5), we can see that *b* is the free acceleration of the end of the rod. Therefore, if *b*>0, lift-off is always possible when *y*=0, *w*=0. But if *b*<0, in equilibrium we would expect a forcing term *F*_*N*_ to maintain the rod on *y*=0. From $\dot{w}=0$, we obtain

2.12 $$F_{N}=-\frac{b}{p_{+}}$$

since *v*>0. If *p*_+_>0, which is always true for *θ*∈(*π*/2,*π*), then *F*_*N*_≥0, in line with (2.11). But if *p*_+_<0, which can happen if *θ*∈(0,*π*/2), then *F*_*N*_<0 in (2.12). Then, *F*_*N*_ is in an *inconsistent* (or *non-existent*) mode. On the other hand, if *b*>0 and *p*_+_<0, then *F*_*N*_>0 in (2.12). At the same time, lift-off is also possible from *y*=0 and hence *F*_*N*_ is in an *indeterminate* (or *non-unique*) mode. It is straightforward to show that *p*_+_(*θ*)<0 requires

2.13 $$\mu >\mu_{P}(\alpha )\equiv \frac{2}{\alpha }\sqrt{1+\alpha }.$$

Then, the Painlevé paradox can occur for *θ*∈(*θ*_1_,*θ*_2_), where

2.14 $$\begin{array}{rl} \theta_{1}(\mu ,\alpha ) & =\arctan\;\frac{1}{2}(\mu \alpha -\sqrt{\mu^{2}\alpha^{2}-4(1+\alpha )})\\ \;\mathrm{and}\;\theta_{2}(\mu ,\alpha ) & =\arctan\;\frac{1}{2}(\mu \alpha +\sqrt{\mu^{2}\alpha^{2}-4(1+\alpha )}). \end{array}\}$$

**Figure 2.**
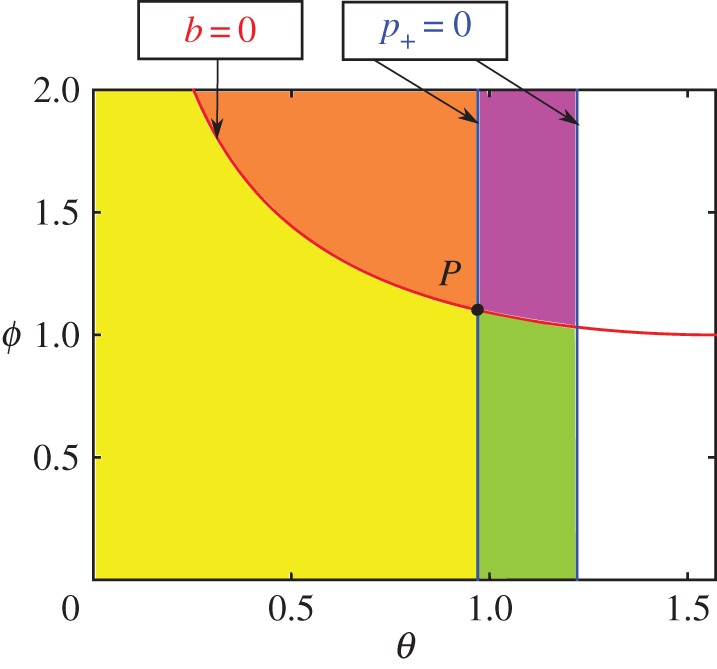
The (*θ*,*ϕ*)-plane for the classical Painlevé problem of figure 1, for *α*=3 and *μ*=1.4. The point *P* has coordinates $\left(\theta_{1},\sqrt{\csc\;\theta_{1}}\right)$, where *θ*_1_ is given in (2.14). In the first quadrant centred on *P*, we have *b*>0, *p*_+_<0, so the dynamics is indeterminate (non-unique). In the second quadrant, *b*>0, *p*_+_>0 and the rod lifts off the rough surface. In the third quadrant, *b*<0, *p*_+_>0 and the rod moves (slips) along the surface. Here, Génot & Brogliato [17] showed that the dynamics cannot cross *p*_+_=0 unless also *b*=0. In the fourth quadrant, *b*<0, *p*_+_<0 and the dynamics is inconsistent (non-existent). Even though the constraint *y*=0 is satisfied, there exists no positive value of *F*_*N*_, contradicting (2.11).

For a uniform rod with *α*=3, we have *μ*_*P*_(3)=4/3. For *α*=3 and *μ*=1.4, the dynamics can be summarized^2^ in the (*θ*,*ϕ*)-plane, as in figure 2. Along *θ*=*θ*_1_,*θ*_2_, we have *p*_+_(*θ*)=0. These lines intersect the curve *b*(*θ*,*ϕ*)=0 at four points: $\phi_{1,2}^{\pm }=\pm \sqrt{\csc\;\theta_{1,2}}$. Génot & Brogliato [17] showed that the point $P:(\theta ,\phi )=(\theta_{1},\sqrt{\csc\;\theta_{1}})$ is the most important and analysed the local dynamics around it. The rigid body equations (2.1) are unable to resolve the dynamics in the third and fourth quadrants. So, we regularize these equations using compliance.

### (b) Compliance-based method

We assume that there is compliance at the point *A* between the rod and the surface, when they are in contact (figure 1). Following [21,28], we assume that there are small excursions into *y*<0. Then we require that the nonnegative normal force *F*_*N*_(*y*,*w*) is a PWS function of (*y*,*w*):

2.15 $$F_{N}(y,w)=[f(y,w)]\equiv \left\{ \begin{array}{cl} 0 & \text{for}\;y>0\\ \max\{f(y,w),0\} & \text{for}\;y\leq 0, \end{array}\right.$$

where the operation [⋅] is defined by the last equality and *f*(*y*,*w*) is assumed to be a smooth function of (*y*,*w*) satisfying ∂_*y*_*f*<0,∂_*w*_*f*<0. The quantities −∂_*y*_*f*(0,0) and −∂_*w*_*f*(0,0) represent a (scaled) spring constant and damping coefficient, respectively. We are interested in the case when the compliance is very large, so we introduce a small parameter *ϵ* as follows:

2.16 $$\partial_{y}f(0,0)=-\epsilon^{-2}\;\mathrm{and}\;\partial_{w}f(0,0)=-\epsilon^{-1}\delta .$$

This choice of scaling [21,28] ensures that the critical damping coefficient (*δ*_crit_=2 in the classical Painlevé problem) is independent of *ϵ*. Our analysis can handle any *f* of the form *f*(*y*,*w*)=*ϵ*^−1^*h*(*ϵ*^−1^*y*,*w*) with

2.17 $$h(\hat{y},w)=-\hat{y}-\delta w+O({\left(\hat{y}+w\right)}^{2}).$$

But, to obtain our quantitative results, we truncate (2.17) and consider the linear function

2.18 $$h(\hat{y},w)=-\hat{y}-\delta w,$$

so that

2.19 $$F_{N}(y,w)=\epsilon^{-1}[-\epsilon^{-1}y-\delta w].$$

In what follows, the first equation in (2.5) will play no role, so we drop it from now on. Then we combine the remaining five equations in (2.5) with (2.15) and (2.16) to give the following set of governing equations that we will use in the sequel:

2.20 $$\begin{array}{rl} \dot{y} & =w,\;\dot{w}=b(\theta ,\phi )+p_{\pm }(\theta )\epsilon^{-1}[-\epsilon^{-1}y-\delta w],\\ \dot{\theta } & =\phi ,\;\dot{\phi }=c_{\pm }(\theta )\epsilon^{-1}[-\epsilon^{-1}y-\delta w]\\ \;\text{and}\;\dot{v} & =a(\theta ,\phi )+q_{\pm }(\theta )\epsilon^{-1}[-\epsilon^{-1}y-\delta w], \end{array}\}$$

For *ϵ*>0, this is a well-defined Filippov system. The slipping region (2.7) and the Filippov vector-field (2.8) are obtained by replacing *F*_*N*_ in these expressions with the square bracket *ϵ*^−1^[−*ϵ*^−1^*y*−*δw*] (see also lemma 4.10).

## 3. Main results

We now present the main results of our paper, theorems 3.1 and 3.2. Theorem 3.1 shows that, if the rod starts in the fourth quadrant of figure 2, it undergoes (regularized) IWC for a time of $O(\epsilon \;\ln\;\epsilon^{-1})$. The same theorem also gives expressions for the resulting vertical velocity of the rod in terms of the compliance damping and initial horizontal velocity and orientation of the rod.

**Theorem 3.1.**

*Consider an initial condition*

3.1 $$(y,w,\theta ,\phi ,v)=(0,O(\epsilon ),\theta_{0},\phi_{0},v_{0}),\;v_{0}>0,$$

*within the region of inconsistency* (*non-existence*) *where*

3.2 $$p_{+}(\theta_{0})<0,\;b(\theta_{0},\phi_{0})<0,$$

*and q*_+_(*θ*_0_)<0, *q*_−_(*θ*_0_)>0, *a*≠0. *Then the forward flow of* (*3.1*) *under* (*2.20*) *returns to* {(*y,w,θ,ϕ,v*)|*y*=0} *after a time* $O(\epsilon \;\ln\;\epsilon^{-1})$ *with*

3.3 $$\begin{array}{rl} w & =e(\delta ,\theta_{0})v_{0}+o(1),\;\theta =\theta_{0}+o(1),\\ \;\mathrm{and}\;\phi & =\phi_{0}+\left\{-\frac{c_{+}(\theta_{0})}{q_{+}(\theta_{0})}+\frac{S_{\phi }(\theta_{0})}{S_{w}(\theta_{0})}\left(e(\delta ,\theta_{0})+\frac{p_{+}(\theta_{0})}{q_{+}(\theta_{0})}\right)\right\}v_{0}+o(1),\;v=o(1), \end{array}\}$$

*as* ϵ→0*. During this time* y=O(ϵ), w=O(1) *so that* $F_{N}=O(\epsilon^{-1})$. *The function e*(*δ,θ*_0_), *given in* (*4.30*), *is smooth and monotonic in δ and has the following asymptotic expansions:*

3.4 $$e(\delta ,\theta_{0})=\frac{p_{-}(\theta_{0})-p_{+}(\theta_{0})}{q_{-}(\theta_{0})p_{+}(\theta_{0})-q_{+}(\theta_{0})p_{-}(\theta_{0})}\delta^{-2}(1+O(\delta^{-2}\;\ln\;\delta^{-1}))\;\text{for}\;\delta \gg 1$$

*and*

3.5 $$\begin{array}{rl} e(\delta ,\theta_{0}) & =\sqrt{\frac{p_{+}(\theta_{0})(p_{-}(\theta_{0})-p_{+}(\theta_{0}))}{q_{+}(\theta_{0})(q_{-}(\theta_{0})-q_{+}(\theta_{0}))}}\\ & \;\times \left(1-\frac{\sqrt{S_{w}(\theta_{0})}}{2}\left(\pi -\text{arctan}\left(\sqrt{-\frac{S_{w}(\theta_{0})}{p_{+}(\theta_{0})}}\right)\right)\delta +O(\delta^{2})\right)\;\mathrm{for}\;\delta \ll 1. \end{array}$$

Theorem 3.2 is similar to theorem 3.1, but now the rod starts in the first quadrant of figure 2. This theorem also gives an exact formula for initial conditions that separate (regularized) IWC and lift-off.

**Theorem 3.2.**

*Consider an initial condition*

3.6 $$(y,w,\theta ,\phi ,v)=(0,\epsilon w_{10},\theta_{0},\phi_{0},v_{0})\;\mathrm{and}\;w_{10}<w_{1*}\equiv -\lambda_{-}(\theta_{0})\frac{b(\theta_{0},\phi_{0})}{p_{+}(\theta_{0})}<0,$$

*with* λ_−_ *defined in* (*4.6*), *within the region of indeterminacy* (*non-uniqueness*) *where*

3.7 $$p_{+}(\theta_{0})<0\;\mathrm{and}\;b(\theta_{0},\phi_{0})>0,$$

*and q*_+_(*θ*_0_)<0, *q*_−_(*θ*_0_)>0, *a*≠0. *Then the conclusions of theorem 3.1, including expressions* (*3.3*)–(*3.5*), *still hold true as* ϵ→0*. For w*_10_>*w*_1*_ *lift-off occurs directly after a time* O(ϵ) *with* w=O(ϵ)*. During this period,* $y=O(\epsilon^{2})$*, so* $F_{N}=O(1)$.

**Remark 3.3.**

These two theorems have not appeared before in the literature. In the rigid-body limit (ϵ→0), we recover IWC in both cases. Previous authors have not carried out the ‘very difficult’ calculation [28], performed numerical calculations [6,21] or given a range of estimates for the time of (regularized) IWC in the *absence* of damping [24]. We give exact and asymptotic expressions for key quantities as well as providing a geometric interpretation of our results, for a large class of rigid bodies, in the presence of a large class of normal forces, as well as giving a precise estimate for the time of (regularized) IWC, all in the presence of both stiffness and damping. Note that we are not attempting to describe all the dynamics around *P*. There is a canard connecting the third quadrant with the first, and the analysis of it is exceedingly complicated [29] due to fast oscillatory terms. Instead, we follow [24] and consider that the rod dynamics starts in a configuration with *p*_+_(*θ*_0_)<0.

## 4. Proof of theorem 3.1: impact without collision in the inconsistent case

**Figure 3.**
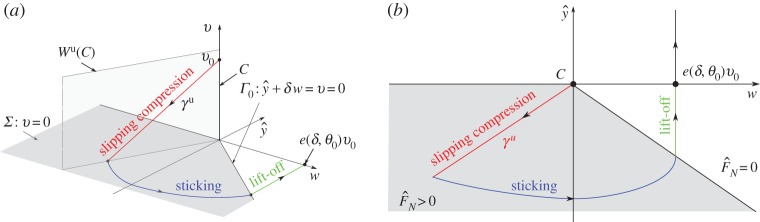
The limit ϵ→0 shown using (*a*) the $\left(w,\hat{y},v\right)$-variables and (*b*) a projection onto the $\left(w,\hat{y}\right)$-plane. The *slipping compression* phase, shown in red, where $\hat{y}$, *w* and *v*>0 all decrease, is described geometrically by an unstable manifold *γ*^u^ (4.5) of a critical set *C*, given in (4.4). It ends on the switching manifold *Σ*. The subsequent *sticking* phase (in blue) is described by Filippov [4]. It ends along *Γ*_0_. From there, the *lift-off* phase (in green) occurs and we return to $\hat{y}=0$. In both figures, the grey region is where ${\hat{F}}_{N}>0$.

The proof of theorem 3.1 is divided into three phases, illustrated in figure 3. These phases are a generalization of the phases of IWC in its rigid-body formulation [15].

— Slipping compression (§4b): During this phase, *y*, *w* and *v* all decrease. The dynamics follow an unstable manifold *γ*^u^ of a set of critical points *C*, given in (4.4) below, as ϵ→0. Along *γ*^u^ the normal force $F_{N}=O(\epsilon^{-1})$ and *v* will therefore quickly decrease to 0. Mathematically, this part is complicated by the fact that the initial condition (3.1) belongs to the critical set *C* as ϵ→0.
— Sticking (§4c): Since $F_{N}=O(\epsilon^{-1})$ and *q*_+_*q*_−_<0, the rod will stick with *v*≡0. During this phase, $\ddot{y}=\dot{w}>0$ and eventually sticking ends with *F*_*N*_=0 as ϵ→0.
— Lift-off (§4d): In the final phase *F*_*N*_=0, lift-off occurs and the system eventually returns to *y*=0.

### (a) Slow–fast setting: initial scaling

Before we consider the first phase of IWC, we apply the scaling

4.1 $$y=\epsilon \hat{y},$$

also used in [21,28], which brings the two terms in (2.19) to the same order. Now let

4.2 $${\hat{F}}_{N}(\hat{y},w)\equiv \epsilon F_{N}(\epsilon \hat{y},w)=[-\hat{y}-\delta w].$$

Equations (2.20) then read

4.3 $$\begin{array}{rl} {\hat{y}}^{\prime } & =w,\;w^{\prime }=\epsilon b(\theta ,\phi )+p_{\pm }(\theta ){\hat{F}}_{N}(\hat{y},w),\\ \theta^{\prime } & =\epsilon \phi ,\;\phi^{\prime }=c_{\pm }(\theta ){\hat{F}}_{N}(\hat{y},w)\\ \;\mathrm{and}\;v^{\prime } & =\epsilon a(\theta ,\phi )+q_{\pm }(\theta ){\hat{F}}_{N}(\hat{y},w), \end{array}\}$$

with respect to the *fast time* *τ*=*ϵ*^−1^*t*, where ()′=d/d*τ*. This is a slow–fast system in non-standard form [28]. Only *θ* is truly slow whereas $\left(\hat{y},w,\phi ,v\right)$ are all fast. But the set of critical points

4.4 $$C=\{(\hat{y},w,\theta ,\phi ,v)|\hat{y}=0,w=0\},$$

for *ϵ*=0 is just three-dimensional. System (4.3) is PWS [26,27]. We now show that (4.3)_+_ contains stable and unstable manifolds *γ*^s,u^ when the equivalent rigid-body equations exhibit a Painlevé paradox, when *p*_+_(*θ*_0_)<0. The saddle structure of *C* within the fourth quadrant has been recognized before [21–23].

**Proposition 4.1.**

*Consider system* (*4.3*)_+_ *with ϵ*=0. *Then for p*_+_(*θ*_0_)<0, *there exist smooth stable and unstable sets γ*^s,u^(*θ*_0_,*ϕ*_0_,*v*_0_), *respectively, of* $(\hat{y},w,\theta ,\phi ,v)=(0,0,\theta_{0},\phi_{0},v_{0})\in C$ *contained within* ${\hat{F}}_{N}\geq 0$ *given by*

4.5 γs,u(θ0,ϕ0,v0)={12(y^,w,θ,ϕ,v)| w=λ∓y^, θ=θ0, ϕ=ϕ0−c+(θ0)λ∓−1[1+δλ∓]y^,v=v0+q+(θ0)p+(θ0)λ∓(θ0)y^, y^≤0},

*with* $\lambda_{\mp }(\theta_{0})≶0$ *given in* (*4.6*).

**Proof.**

Consider the smooth system, (4.3)${\;}_{{\hat{F}}_{N}=-\hat{y}-\delta w}$, obtained from (4.3) by setting ${\hat{F}}_{N}=-\hat{y}-\delta w$ with *ϵ*=0. The linearization of (4.3)${\;}_{{\hat{F}}_{N}=-\hat{y}-\delta w}$ about a point in *C* with *ϵ*=0 then only has two non-zero eigenvalues:

4.6 $$\lambda_{\pm }(\theta )=-\frac{\delta p_{+}(\theta )}{2}\pm \frac{1}{2}\sqrt{\delta^{2}p_{+}{\left(\theta \right)}^{2}-4p_{+}(\theta )},$$

satisfying

4.7 $$\lambda_{\pm }^{2}=-p_{+}(\theta )(1+\delta \lambda_{\pm }).$$

For *p*_+_(*θ*)<0, we have λ_−_<0<λ_+_. The eigenvectors associated with λ_±_ are *v*_±_=(1,λ_±_,0,(*c*_+_/*p*_+_(*θ*))λ_±_,(*q*_+_/*p*_+_(*θ*))λ_±_)^*T*^. Therefore, the smooth system (4.3)${\;}_{{\hat{F}}_{N}=-\hat{y}-\delta w,\epsilon =0}$ has a (stable and unstable) manifold *γ*^s,u^ tangent to *v*_∓_ at $\left(\hat{y},w,\theta ,\phi ,v)=(0,0,\theta_{0},\phi_{0},v_{0}\right)$. But then for $\hat{y}\leq 0$, we have ${\hat{F}}_{N}(\hat{y},\lambda_{\pm }\hat{y})=-(1+\delta \lambda_{\pm })\hat{y}=(\lambda_{\pm }^{2}/p_{+}(\theta ))\hat{y}\geq 0$, by (4.7). Hence, the restrictions of *γ*^s,u^ in (4.5) to $\hat{y}\leq 0$ are (stable and unstable) sets of *C* for the PWS system (4.3)${\;}_{{\hat{F}}_{N}=[-\hat{y}-\delta w],\epsilon =0}$. ▪

**Remark 4.2.**

For the smooth system (4.3)${\;}_{{\hat{F}}_{N}=-\hat{y}-\delta w}$, the critical manifold *C* perturbs by Fenichel’s theory [30–32] to a smooth slow manifold *C*_*ϵ*_, being $C^{\infty }$ O(ϵ)-close to *C* for 0<*ϵ*≪1. A simple calculation shows that $C_{\epsilon }:\;\hat{y}=\epsilon (b(\theta ,\phi )/p_{+}(\theta ))(1+O(\epsilon ))$, $w=O(\epsilon^{2})$. Since *b*(*θ*,*ϕ*)<0 in this case, $C_{\epsilon }\subset \{\hat{y}>0\}$ for *ϵ* sufficiently small. Therefore, the manifold *C*_*ϵ*_ is only invariant for the smooth system (4.3)${\;}_{{\hat{F}}_{N}=-\hat{y}-\delta w}$. It is an artefact for the PWS system (4.3)${\;}_{{\hat{F}}_{N}=[-\hat{y}-\delta w]}$ since the square bracket vanishes for $\hat{y}>0$, by (2.15).

**Remark 4.3.**

Our arguments are geometrical and rely on hyperbolic methods of dynamical systems theory only. Therefore, the results remain unchanged qualitatively if we replace the piecewise linear ${\hat{F}}_{N}$ in (4.2) with the nonlinear version ${\hat{F}}_{N}(\hat{y},w)=[h(\hat{y},w)]$, where $h(\hat{y},w)=-\hat{y}-\delta w+O({\left(\hat{y}+w\right)}^{2})$ as in (2.17), having (2.18) as its linearization about $\hat{y}=w=0$. We would obtain again a saddle-type critical set *C* with nonlinear (stable and unstable) manifolds *γ*^s,u^.

Following the initial scaling (4.1) of this section, we now consider the three phases of IWC.

### (b) Slipping compression

The first phase of the regularized IWC: *slipping compression* ends on the *switching manifold*

4.8 $$\Sigma =\{(\hat{y},w,\theta ,\phi ,v)|v=0\},$$

shown in figure 3*a*. Proposition 4.4 describes the intersection of the forward flow of initial conditions (3.1) with *Σ*.

**Proposition 4.4.**

*The forward flow of the initial conditions* (*3.1*) *under* (*4.3*) *intersects Σ in*

4.9 γu∩Σ+o(1)≡{12(y^,w,θ,ϕ,v)∈Σ| y^=−p+(θ0)q+(θ0)λ+(θ0)v0+o(1), w=−p+(θ0)q+(θ0)v0+o(1),θ=θ0+o(1), ϕ=ϕ0−c+(θ0)q+(θ0)v0+o(1)},

*as* ϵ→0.

**Remark 4.5.**

The *o*(1)-term in (4.9) is $O(\epsilon^{c})$ for any *c*∈(0,1) (see also lemma 4.8).

#### (i) Proof of proposition 4.4

We prove proposition 4.4 using Fenichel’s normal form theory [33]. But since (4.3)${\;}_{{\hat{F}}_{N}=[-\hat{y}-\delta w]}$ with *v*>0 is PWS, care must be taken. There are at least two ways to proceed. One way is to consider the smooth system (4.3)${\;}_{{\hat{F}}_{N}=-\hat{y}-\delta w}$, then rectify *C*_*ϵ*_ by straightening out its stable and unstable manifolds. Then, (4.3)${\;}_{{\hat{F}}_{N}=-\hat{y}-\delta w}$ will be a standard slow–fast system to which Fenichel’s normal form theory applies. Subsequently, one would then have to ensure that conclusions based on the smooth (4.3)${\;}_{{\hat{F}}_{N}=-\hat{y}-\delta w}$ also extend to the PWS system (4.3)${\;}_{{\hat{F}}_{N}=[-\hat{y}-\delta w]}$. One way to do this is to consider the following scaling

4.10 $$\kappa_{1}:\;\hat{y}=r_{1}{\hat{y}}_{1},\;w=r_{1}w_{1},\;\epsilon =r_{1},$$

zooming in on *C* at $\hat{y}=0,w=0$. In terms of the original variables, $y=\epsilon^{2}{\hat{y}}_{1}$, *w*=*ϵw*_1_. Both the scalings $\left(\hat{y},w\right)$ and $\left({\hat{y}}_{1},w_{1}\right)$ have appeared in the literature [6,21,28].

In this paper, we follow another approach (basically reversing the process described above) which works more directly with the PWS system. Therefore, in §4b(ii), we study the scaling (4.10) first. We will show that the $\left({\hat{y}}_{1},w_{1}\right)$-system contains important geometry of the PWS system (significant, for example, for the separation of initial conditions in theorem 3.2). Then in §4b(iii), we connect the ‘small’ $\left(\hat{y}=O(\epsilon ),w=O(\epsilon )\right)$ described by (4.10) with the ‘large’ ($\hat{y}=O(1),w=O(1)$) in (4.3) by considering coordinates described by the following transformation:

4.11 $$\kappa_{2}:\;\hat{y}=-r_{2},\;w=r_{2}w_{2},\;\epsilon =r_{2}\epsilon_{2}.$$

For *y*_1_<0, we have the following coordinate change *κ*_21_ between *κ*_1_ and *κ*_2_:

4.12 $$\kappa_{21}:\;r_{2}=-r_{1}y_{1},\;w_{2}=-w_{1}y_{1}^{-1},\;\epsilon_{2}=-y_{1}^{-1}.$$

The coordinates in *κ*_2_ (4.11) appear as a *directional chart* obtained by setting $\bar{y}=-1$ in the blowup transformation $\left(r,\bar{\hat{y}},\bar{w},\bar{\epsilon })\mapsto (\hat{y},w,\epsilon \right)$ given by^3^

4.13 $$\hat{y}=r\bar{\hat{y}},\;w=r\bar{w},\;\epsilon =r\bar{\epsilon },\;r\geq 0,\;(\bar{\hat{y}},\bar{w},\bar{\epsilon })\in S^{2}=\{(\bar{\hat{y}},\bar{w},\bar{\epsilon })|{\bar{\hat{y}}}^{2}+{\bar{w}}^{2}+{\bar{\epsilon }}^{2}=1\}.$$

The blowup is chosen so that the zoom in (4.10) coincides with the *scaling chart* obtained by setting $\bar{\epsilon }=1$. The blowup transformation *blows up* *C* to $\bar{C}:\;r=0,\;(\bar{\hat{y}},\bar{w},\bar{\epsilon })\in S^{2}$ a space $(\theta ,\phi ,v)\in R^{3}$ of spheres.^4^

The main advantage of our approach is that in chart *κ*_2_ we can focus on $\bar{C}\cap \{{\bar{\hat{y}}}^{-1}\bar{w}>-\delta^{-1},\bar{\hat{y}}<0\}$ (or simply *w*_2_<*δ*^−1^ in (4.11)) of $\bar{C}$, the grey area in figure 3, where

4.14 $$r^{-1}{\hat{F}}_{N}(\hat{y},w)=[-\bar{\hat{y}}-\delta \bar{w}]=-\bar{\hat{y}}(1+\delta {\bar{\hat{y}}}^{-1}\bar{w})>0,$$

and the system will be smooth. This enables us to apply Fenichel’s normal form theory [33] there. All the necessary patching for the PWS system is done independently in the scaling chart *κ*_1_.

#### (ii) Chart *κ*_1_

Let ${\hat{F}}_{N,1}({\hat{y}}_{1},w_{1})=\epsilon^{-1}{\hat{F}}_{N}(\epsilon {\hat{y}}_{1},\epsilon w_{1})=[-{\hat{y}}_{1}-\delta w_{1}].$ Then applying chart *κ*_1_ in (4.10) to the non-standard slow–fast system (4.3) gives the following equations:

4.15 $$\begin{array}{rl} {\hat{y}}_{1}^{\prime } & =w_{1},\;w_{1}^{\prime }=b(\theta ,\phi )+p_{+}(\theta ){\hat{F}}_{N,1}({\hat{y}}_{1},w_{1}),\\ \theta^{\prime } & =\epsilon \phi ,\;\phi^{\prime }=\epsilon c_{+}(\theta ){\hat{F}}_{N,1}({\hat{y}}_{1},w_{1})\\ \;\mathrm{and}\;v^{\prime } & =\epsilon (a(\theta ,\phi )+q_{+}(\theta ){\hat{F}}_{N,1}({\hat{y}}_{1},w_{1})). \end{array}\}$$

**Figure 4.**
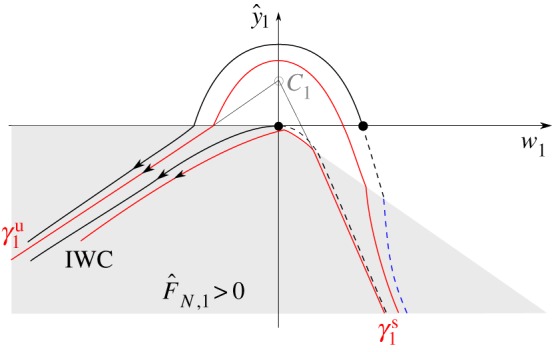
Phase portrait (4.15)_*ϵ*=0_ for *b*<0. Theorem 3.1 considers initial conditions on the *w*_1_-axis. The critical set *C*_1_ of (4.15)${\;}_{{\hat{F}}_{N,1}=-{\hat{y}}_{1}-\delta w_{1},\epsilon =0}$ is an artefact of the PWS system. The grey region is now where ${\hat{F}}_{N,1}>0$. Orbit segments outside this region are parabolas. Dashed lines indicate backward orbits, from initial conditions on the *w*_1_-axis. A similar figure appears in [6].

The above equation is a slow–fast system in standard form: $\left({\hat{y}}_{1},w_{1}\right)$ are fast variables, whereas (*θ*,*ϕ*,*v*) are slow variables. By assumption (3.2) of theorem 3.1, *b*<0, *p*_+_<0 and so, since ${\hat{F}}_{N,1}({\hat{y}}_{1},w_{1})\geq 0$, we have *w*_1_′<0 in (4.15). Hence, there exists no critical set for the PWS system (4.15)_*ϵ*=0_. (The critical set $C_{1}=\{({\hat{y}}_{1},w_{1},\theta ,\phi ,v)|{\hat{y}}_{1}=b(\theta ,\phi )/p_{+}(\theta ),w_{1}=0\}$ of the *smooth* system (4.15)${\;}_{{\hat{F}}_{N,1}=-{\hat{y}}_{1}-\delta w_{1},\epsilon =0}$ lies within ${\hat{y}}_{1}>0$. It is therefore an artefact of the PWS system, as illustrated in figure 4 (recall also remark 4.2).)

The unstable manifold $\gamma_{1}^{u}$ of *C*_1_ in the smooth system (4.15)${\;}_{{\hat{F}}_{N,1}=-{\hat{y}}_{1}-\delta w_{1}}$ is given by $w_{1}=\lambda_{+}(\theta_{0})({\hat{y}}_{1}-b(\theta_{0},\phi_{0})/p_{+}(\theta_{0}))$ and its restriction

4.16 $$\gamma_{1}^{u}(\theta_{0},\phi_{0},v_{0})=\left\{({\hat{y}}_{1},w_{1},\theta_{0},\phi_{0},v_{0})|\;w_{1}=\lambda_{+}(\theta_{0})\left({\hat{y}}_{1}-\frac{b(\theta_{0},\phi_{0})}{p_{+}(\theta_{0})}\right),\;{\hat{y}}_{1}\leq 0\right\},$$

to the subset ${\hat{y}}_{1}\leq 0$, *w*_1_≤0 where ${\hat{F}}_{N,1}\geq 0$, is locally invariant for the PWS system (4.15)${\;}_{{\hat{F}}_{N,1}=[-{\hat{y}}_{1}-\delta w_{1}]}$. In chart *κ*_1_, initial conditions (3.1) now become

4.17 $$({\hat{y}}_{1},w_{1},\theta ,\phi ,v)=(0,O(1),\theta_{0},\phi_{0},v_{0}).$$

**Lemma 4.6.**

*Consider* $\Lambda_{1}=\{({\hat{y}}_{1},w_{1},\theta ,\phi ,v)|{\hat{y}}_{1}=-\nu^{-1}\}$ *with ν*>0 *small. Then, the forward flow of* (*4.17*) *under* (*4.15*) *intersects Λ*_1_ *in*

4.18 $$z_{1}(\epsilon )\equiv (-\nu^{-1},w_{1c}(\nu )+O(\epsilon ),\theta_{0}+O(\epsilon ),\phi_{0}+O(\epsilon ),v_{0}+O(\epsilon )),$$

*where* $w_{1c}(\nu )=-\lambda_{+}\nu^{-1}(1+o(1)),\;\nu \to 0$.

**Proof.**

Consider the layer problem (4.15)_*ϵ*=0_. Since *b*<0, initial conditions (4.17) with *w*_1_>0 return to ${\hat{y}}_{1}=0$ with *w*_1_<0, see figure 4. Therefore, we consider *w*_1_(0)≤0 subsequently. From λ_−_<0<λ_+_, it then follows that the solution remains within ${\hat{F}}_{N,1}>0$ for *τ*>0 for *ϵ*=0. The problem is therefore linear. The remaining details of the proof are straightforward and hence omitted. ▪

For *ϵ*>0, the variables (*ϕ*,*v*) will vary by O(1)-amount as ${\hat{y}}_{1},w_{1}\to -\infty$. But the variables (*ϕ*,*v*) are fast in (4.3) and slow in (4.15). To describe this transition, we change to chart *κ*_2_.

#### (iii) Chart *κ*_2_

Writing the non-standard slow–fast PWS system (4.3)${\;}_{{\hat{F}}_{N}=[-\hat{y}-\delta w]}$ in chart *κ*_2_, given by (4.11), gives the following smooth (as anticipated by (4.14)) system:

4.19 $$\begin{array}{rl} \epsilon_{2}^{\prime } & =\epsilon_{2}w_{2},\;w_{2}^{\prime }=\epsilon_{2}b(\theta ,\phi )+p_{+}(\theta )(1-\delta w_{2})+w_{2}^{2},\\ \theta^{\prime } & =\epsilon \phi ,\;\phi^{\prime }=c_{+}(\theta )r_{2}(1-\delta w_{2}),\\ \;v^{\prime } & =\epsilon a(\theta ,\phi )+q_{+}(\theta )r_{2}(1-\delta w_{2})\;\text{and}\;r_{2}^{\prime }=-r_{2}w_{2}, \end{array}\}$$

on the box *U*_2_={(*ϵ*_2_,*w*_2_,*θ*,*ϕ*,*v*,*r*_2_)|*ϵ*_2_∈[0,*ν*],*w*_2_∈[−λ_+_−*ρ*,−λ_+_+*ρ*],*r*_2_∈[0,*ν*]}, for *ρ*>0 sufficiently small (so that *w*_2_<*δ*^−1^) and *ν* as above. Notice that *z*_1_(*ϵ*) from (4.18) in chart *κ*_2_ becomes

4.20 $$z_{2}(\epsilon )\equiv \kappa_{21}(z_{1}(\epsilon )):\;r_{2}=\epsilon \nu^{-1},\;w_{2}=w_{1c}\nu +O(\epsilon ),\;\epsilon_{2}=\nu ,$$

using (4.12). Clearly, *z*_2_(*ϵ*)∈*κ*_21_(*Λ*_1_)⊂*U*_2_, *κ*_21_(*Λ*_1_) being the face of the box *U*_2_ with *ϵ*_2_=*ν*. For simplicity, we will write subsets such as {(*ϵ*_2_,*w*_2_,*θ*,*ϕ*,*v*,*r*_2_)∈*U*_2_|⋯ } by {*U*_2_|⋯ }.

**Lemma 4.7.**

*The set M*_2_={*U*_2_| *r*_2_=0,*ϵ*_2_=0,*w*_2_=−λ_+_} *is a set of critical points of* (*4.19*). *Linearization around M*_2_ *gives only three non-zero eigenvalues* −λ_+_<0, λ_−_−λ_+_<0, λ_+_>0, *and so M*_2_ *is of saddle-type. The stable manifold is W*^s^(*M*_2_)={*U*_2_|*r*_2_=0} *while the unstable manifold is W*^u^(*M*_2_)={*U*_2_|*ϵ*_2_=0,*w*_2_=−λ_+_}. *In particular, the one-dimensional unstable manifold γ*^u^_2_(*θ*_0_,*ϕ*_0_,*v*_0_)⊂*W*^u^(*M*_2_) *of the base point* (*ϵ*_2_,*w*_2_,*θ*,*ϕ*,*v*,*r*_2_)=(0,0,*θ*_0_,*ϕ*_0_,*v*_0_,0)∈*M*_2_ *is given by*

4.21 γ2u(θ0,ϕ0,v0)={12U2| w2=−λ+(θ0), θ=θ0, ϕ=ϕ0−c+(θ0)p+(θ0)λ+(θ0)r2,v=v0−q+(θ0)p+(θ0)λ+(θ0)r2, r2≥0, ϵ2=0}.

**Proof.**

The first two statements follow from straightforward calculation. For $\gamma_{2}^{u}(\theta_{0},\phi_{0},v_{0})$, we restrict to the invariant set *ϵ*_2_=0, *w*_2_=−λ_+_ and solve the resulting reduced system. ▪

Notice that the set *γ*^u^_2_(*θ*_0_,*ϕ*_0_,*v*_0_) is just *γ*^u^(*θ*_0_,*ϕ*_0_,*v*_0_) in (4.5) written in chart *κ*_2_ for *ϵ*_2_=0. Furthermore, note that *z*_2_(0)⊂*W*^s^(*M*_2_). In the subsequent lemma, we follow *z*_2_(*ϵ*)⊂{*ϵ*_2_=*ν*} up until *r*_2_=*ν*, with *ν* sufficiently small, by applying Fenichel’s normal form theory.

**Lemma 4.8.**

*Let c*∈(0,1) *and set Λ*_2_={*U*_2_|*r*_2_=*ν*}. *Then as* ϵ→0, *for ν and ρ sufficiently small, the forward flow of z*_2_(*ϵ*) *in* (*4.20*) *intersects Λ*_2_ *in*

4.22 $$\begin{array}{rl} & \{\Lambda_{2}|\;w_{2}=-\lambda_{+}+O(\epsilon^{c}),\;\theta =\theta_{0}+O(\epsilon \;\ln\;\epsilon^{-1}),\;\phi =\phi_{0}-\frac{c_{+}(\theta_{0})}{p_{+}(\theta_{0})}\lambda_{+}(\theta_{0})\nu +O(\epsilon^{c}),\\ & \;v=v_{0}-\frac{q_{+}(\theta_{0})}{p_{+}(\theta_{0})}\lambda_{+}(\theta_{0})\nu +O(\epsilon^{c})\}. \end{array}$$

**Proof.**

By Fenichel’s normal form theory, we can straighten out stable and unstable fibres.

**Lemma 4.9.**

*For ν and ρ sufficiently small, then within U*_2_ *there exists a smooth transformation* $\left(\epsilon_{2},w_{2},\phi ,v,r_{2})\mapsto (\tilde{\phi },\tilde{v}\right)$ *satisfying*

4.23 $$\begin{array}{rl} \tilde{\phi } & =\phi +\frac{c_{+}(\theta )}{p_{+}(\theta )}\lambda_{+}(\theta )r_{2}+O(r_{2}(w_{2}+\lambda_{+}))\\ \;\mathrm{and}\;\tilde{v} & =v+\frac{q_{+}(\theta )}{p_{+}(\theta )}\lambda_{+}(\theta )r_{2}+O(r_{2}(w_{2}+\lambda_{+})+\epsilon ), \end{array}\}$$

*which transforms* (*4.19*) *into*

4.24 $$\begin{array}{rl} \epsilon_{2}^{\prime } & =\epsilon_{2}w_{2},\;w_{2}^{\prime }=\epsilon_{2}b(\theta ,\tilde{\phi })+p_{+}(\theta )(1-\delta w_{2})+w_{2}^{2}+O(\epsilon ),\\ \theta^{\prime } & =\epsilon \tilde{\phi },\;{\tilde{\phi }}^{\prime }=0,\\ {\tilde{v}}^{\prime } & =0\;\mathrm{and}\;r_{2}^{\prime }=-r_{2}w_{2}. \end{array}\}$$

**Proof.**

Replace *r*_2_ by *νr*_2_ in (4.19) and consider *ν* small. Using *ϵ*=*ϵ*_2_*r*_2_, this brings the system into a classical slow–fast system for 0<*ν*≪1, where (*ϵ*_2_,*w*_2_,*r*_2_) are fast variables while (*θ*,*ϕ*,*v*) are slow. In particular, *ϵ*_2_=*r*_2_=0, *w*_2_=−λ_+_ is a saddle-type slow manifold for *ν* small. The system is therefore amenable to Fenichel’s normal form theory [33]. The result then follows by returning to the original *r*_2_ and using $\phi =\tilde{\phi }+O(r_{2})$ together with *r*_2_*ϵ*_2_=*ϵ* in the *w*_2_ equation. ▪

To prove lemma 4.8, we then integrate the normal form (4.24) with initial conditions *z*_2_(*ϵ*) from (4.20) from (a reset) time *τ*=0 up to *τ*=*T*, defined implicitly by *r*_2_(*T*)=*ν*. Clearly, $\theta (T)=\theta_{0}+O(\epsilon T)$, $\tilde{\phi }(T)={\tilde{\phi }}_{0}$ and $\tilde{v}(T)={\tilde{v}}_{0}$. Then, from (4.12), Gronwall’s inequality and the fact that 1−λ_−_λ^−1^_+_>1, we find

4.25 $$\begin{array}{rl} T & =\lambda_{+}^{-1}\;\ln\;\epsilon^{-1}(1+o(1))\\ \;\mathrm{and}\;w_{2}(T) & =-\lambda_{+}(1+O(e^{-\lambda_{+}T}+e^{(\lambda_{-}-\lambda_{+})T}+\epsilon ))\\ & =-\lambda_{+}+O(\epsilon^{c(1-\lambda_{-}\lambda_{+}^{-1})}+\epsilon^{c})=-\lambda_{+}+O(\epsilon^{c}), \end{array}\}$$

for *c*∈(0,1). Then, we obtain the expressions for *θ*=*θ*(*T*), *ϕ*=*ϕ*(*T*) and *v*=*v*(*T*) in (4.22) from (4.23) in terms of the original variables. ▪

#### (iv) Completing the proof of proposition 4.4

To complete the proof of proposition 4.4, we then return to (4.3) using (4.11) and integrate initial conditions (4.22) within $\left\{\hat{y}=-r_{2}=-\nu \right\}$, up to the switching manifold *Σ*={*v*=0}, using regular perturbation theory and the implicit function theorem. This gives (4.9), which completes the proof of proposition 4.4.

### (c) Sticking

After the slipping compression phase of the previous section, the rod then sticks on *Σ*, with $\left(\hat{y},w,\theta ,\phi \right)$ given by (4.9). This is a corollary of the following lemma.

**Lemma 4.10.**

*Suppose a*≠0, *q*_+_<0, *q*_−_>0. *Consider the* (*negative*) *function*

$$F(\theta ,\phi )=\left\{ \begin{array}{ll} \frac{a(\theta ,\phi )}{q_{+}(\theta )} & \text{if}\;a>0,\\ \frac{a(\theta ,\phi )}{q_{-}(\theta )} & \text{if}\;a<0. \end{array}\right.$$

*Then there exists a set of visible folds at*

4.26 $$\Gamma_{\epsilon }\equiv \{(\hat{y},w,\theta ,\phi ,v)\in \Sigma |\;\hat{y}+\delta w=\epsilon F(\theta ,\phi )\},$$

*of the Filippov system* (*4.3*), *dividing the switching manifold Σ*:*v*=0 *into* (*stable*) *sticking*: $\Sigma_{s}\equiv \{(\hat{y},w,\theta ,\phi ,v)\in \Sigma |\;\hat{y}+\delta w<\epsilon F(\theta ,\phi )\},$ *and crossing upwards* (*downwards*) *for a*>0 (*a*<0): $\Sigma_{c}\equiv \{(\hat{y},w,\theta ,\phi ,v)\in \Sigma |\;\hat{y}+\delta w>\epsilon F(\theta ,\phi )\}$.

**Proof.**

Simple computations, following [4]; see also proposition 2.1. ▪

The forward motion of (4.9) within *Σ*_*s*_⊂*Σ* for *ϵ*≪1 is therefore subsequently described by the Filippov vector-field (2.8) in proposition 2.1,

4.27 $$\begin{array}{rl} {\hat{y}}^{\prime } & =w,\;w^{\prime }=\epsilon b(\theta ,\phi )+S_{w}(\theta )[-\hat{y}-\delta w],\\ \theta^{\prime } & =\epsilon \phi \;\text{and}\;\phi^{\prime }=S_{\phi }(\theta )[-\hat{y}-\delta w], \end{array}\}$$

here written in terms of $\hat{y}$ and the fast time *τ*, until sticking ends at the visible fold *Γ*_*ϵ*_. Note this always occurs for 0<*ϵ*≪1 since ${\hat{y}}^{\prime \prime }=w^{\prime }>0$, for $[-\hat{y}-\delta w]>0$.

We first focus on *ϵ*=0. From (4.27), *θ*=*θ*_0_, a constant, and

4.28 $$\begin{array}{rl} {\hat{y}}^{\prime } & =w,\\ w^{\prime } & =S_{w}(\theta )[-\hat{y}-\delta w]\\ \;\mathrm{and}\;\phi^{\prime } & =S_{\phi }(\theta )[-\hat{y}-\delta w]. \end{array}\}$$

We now integrate (4.28), using (4.9) for *ϵ*=0 as initial conditions, given by

4.29 $$(\hat{y}(0),w(0),\phi (0))=\left(-\frac{p_{+}(\theta_{0})}{q_{+}(\theta_{0})\lambda_{+}(\theta_{0})}v_{0},-\frac{p_{+}(\theta_{0})}{q_{+}(\theta_{0})}v_{0},\phi_{0}-\frac{c_{+}(\theta_{0})}{q_{+}(\theta_{0})}v_{0}\right),$$

up until the section $\Gamma_{0}:\hat{y}+\delta w=0$ shown in figure 3*a*, where sticking ceases for *ϵ*=0, by lemma 4.10 and (4.26)_*ϵ*=0_. We then obtain a function *e*(*δ*,*θ*_0_)>0 in the following proposition, which relates the horizontal velocity at the start of the slipping compression phase *v*_0_ (3.1) with the values of $\left(\hat{y},w,\phi \right)$ on *Γ*_0_, at the end of the sticking phase.

**Proposition 4.11.**

*There exists a smooth function e*(*δ*,*θ*_0_)>0 *and a time τ*_*s*_>0 *such that* $(\hat{y}(\tau_{s}),w(\tau_{s}),\phi (\tau_{s}))\in \Gamma_{0}$ *with*

$$\begin{array}{rl} \hat{y}(\tau_{s}) & =-\delta e(\delta ,\theta_{0})v_{0},\\ w(\tau_{s}) & =e(\delta ,\theta_{0})v_{0}\\ \;\mathrm{and}\;\phi (\tau_{s}) & =\phi_{0}+\left\{-\frac{c_{+}(\theta_{0})}{q_{+}(\theta_{0})}+\frac{S_{\phi }(\theta_{0})}{S_{w}(\theta_{0})}\left(e(\delta ,\theta_{0})+\frac{p_{+}(\theta_{0})}{q_{+}(\theta_{0})}\right)\right\}v_{0}, \end{array}$$

*where* $\left(\hat{y}(\tau ),w(\tau ),\phi (\tau )\right)$ *is the solution of* (*4.28*) *with initial conditions* (*4.29*). *The function e*(*δ*,*θ*_0_) *is monotonic in δ*: ∂_*δ*_*e*(*δ*,*θ*_0_)<0, *and satisfies* (*3.4*) *and* (*3.5*) *for δ*≫1 *and δ*≪1, *respectively*.

**Proof.**

The existence of *τ*_*s*_ is obvious. Linearity in *v*_0_ follows from (4.29) and the linearity of (4.28) within ${\hat{F}}_{N}>0$. Since $\dot{\hat{y}}=w$, we have *e*>0. The *ϕ* equation follows since *ϕ*′=*w*′*S*_*ϕ*_(*θ*)/*S*_*w*_(*θ*). The monotonicity of *e* as a function *δ* is the consequence of simple arguments in the $\left(w,\hat{y}\right)$-plane using (4.28) and the fact that *w*(0) in (4.29) is independent of *δ* while $\hat{y}(0)={\hat{y}}_{0}(\delta )$ decreases (since λ_+_ is an increasing function of *δ*). To obtain the asymptotics, we first solve (4.28) with $\delta \neq 2/\sqrt{S_{w}(\theta_{0})}$. Simple calculations show that

4.30 $$e(\delta ,\theta_{0})=\frac{\xi_{+}}{\xi_{-}}(\lambda_{+}-\xi_{-})\frac{p_{+}}{q_{+}\lambda_{+}}e^{\xi_{+}\tau_{s}},$$

suppressing the dependency on *θ*_0_ on the right-hand side, where $\xi_{\pm }=-\delta S_{w}/2\pm \frac{1}{2}\sqrt{\delta^{2}S_{w}^{2}-4S_{w}}$ and *τ*_*s*_ is the least positive solution of *e*^(*ξ*_+_−*ξ*_−_)*τ*_*s*_^=*ξ*^2^_−_(λ_+_−*ξ*_+_)/*ξ*^2^_+_(λ_+_−*ξ*_−_). For *δ*≫1, the eigenvalues *ξ*_±_ are real and negative. Hence, $\tau_{s}=1/(\xi_{+}-\xi_{-})\;\ln\;(\xi_{-}^{2}(\lambda_{+}-\xi_{+})/\xi_{+}^{2}(\lambda_{+}-\xi_{-}))$. Now using $\xi_{+}=-S_{w}\delta (1+O(\delta^{-2}),\;\xi_{-}=S_{w}/\xi_{-}=-\delta^{-1}(1+O(\delta^{-1})$, and $\lambda_{+}=-p_{+}\delta (1+O(\delta^{-2})),$ we obtain $\xi_{+}\tau_{s}=O(\delta^{-2}\;\ln\;\delta^{-1}),$ and hence

4.31 $$e(\delta ,\theta_{0})=-\frac{S_{w}-p_{+}}{q_{+}S_{w}}\delta^{-2}(1+O(\delta^{-2}\;\ln\;\delta^{-1})),$$

as δ→∞. For *δ*≪1, *ξ*_±_ are complex conjugated with negative real part. This gives *τ*_*s*_=(2*i*/(*ξ*_+_−*ξ*_−_))(*χ*−*πn*), *χ*=arg((λ_+_−*ξ*_+_)*ξ*^2^_−_)>0, *n*=⌊*χ*/*π*⌋. Using the asymptotics of *ξ*_±_ and λ_+_, we obtain $\tau_{s}=(\pi -\text{arctan}(\sqrt{-S_{w}/p_{+}}))/\sqrt{S_{w}}-\frac{1}{2}\delta (1+O(\delta )),$ and then

4.32 $$e(\delta ,\theta_{0})=-\frac{\sqrt{p_{+}(p_{+}-S_{w})}}{q_{+}}\left(1-\frac{\sqrt{S_{w}}}{2}\left(\pi -\text{arctan}\left(\sqrt{-\frac{S_{w}}{p_{+}}}\right)\right)\delta +O(\delta^{2})\right),$$

as $\delta \to 0^{+}$. Simple algebraic manipulations of (4.31) and (4.32) using (2.9) give the expressions in (3.4) and (3.5). ▪

**Remark 4.12.**

The critical value $\delta =\delta_{\mathrm{crit}}(\theta_{0})\equiv 2/\sqrt{S_{w}(\theta_{0})}$ gives a double root of the characteristic equation. For the classical Painlevé problem, *δ*_crit_(*π*/2)=2, as expected (see §2b).

For 0<*ϵ*≪1, sticking ends along the visible fold at *Γ*_*ϵ*_. We therefore perturb from *ϵ*=0 as follows:

**Proposition 4.13.**

*The forward flow of* (*4.9*) *under the Filippov vector-field* (*4.27*) *intersects the set of visible folds Γ*_*ϵ*_ *o*(1)-*close to the intersection of* (*4.9*)_*ϵ*=0_ *with Γ*_0_ *described in proposition* 4.11.

**Proof.**

Since the *ϵ*=0 system is transverse to *Γ*_0_, we can apply regular perturbation theory and the implicit function theorem to perturb *τ*_*s*_ continuously to *τ*_*s*_+*o*(1). The result then follows. ▪

### (d) Lift-off

Beyond *Γ*_*ϵ*_ we have ${\hat{F}}_{N}\equiv 0$ and lift-off occurs. For *ϵ*=0, we have ${\hat{y}}^{\prime }=w$ and *w*′=*θ*′=*ϕ*′=*v*′=0. By proposition 4.13 and regular perturbation theory, we obtain the desired result in theorem 3.1. In terms of the original (slow) time *t*, it follows that the time of IWC is of order $O(\epsilon \;\ln\;\epsilon^{-1})$ (recall (4.25)). As ϵ→0, IWC occurs instantaneously.

## 5. Proof of theorem 3.2: impact without collision in the indeterminate case

**Figure 5.**
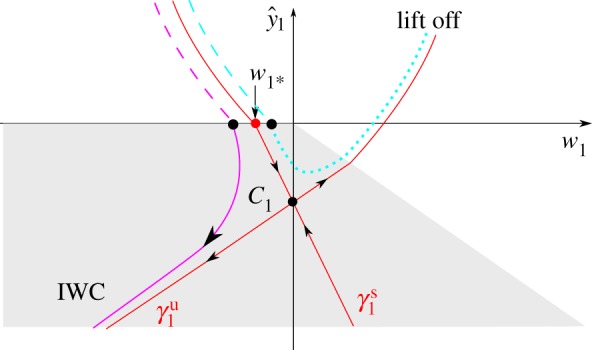
Phase portrait (4.15)_*ϵ*=0_ for *b*>0 (theorem 3.2 in §5). Here, $C_{1}=\{{\hat{y}}_{1}=b/p_{+},w_{1}=0\}$ is a saddle-type critical manifold for the PWS system, $\gamma_{1}^{u}$ is given by (4.16), $\gamma_{1}^{s}$ by (5.1) and *w*_1*_ by (5.2). As in figure 4, the grey region is where ${\hat{F}}_{N,1}>0$. Orbit segments outside this region are parabolas. Dashed lines indicate backward orbits, from initial conditions on the *w*_1_-axis. A similar figure appears in [6].

Here, by assumption (3.7), we have *b*>0. Therefore, we have, using *p*_+_<0, that $C_{1}=\{{\hat{y}}_{1}=b/p_{+},w_{1}=0\}\subset \{{\hat{y}}_{1}<0\}$ is a critical set of (4.15)_*ϵ*=0_; see also figure 5. The stable manifold of *C*_1_∩{*θ*=*θ*_0_,*ϕ*=*ϕ*_0_,*v*=*v*_0_} within $\hat{y}\leq 0$ is

5.1 $$\gamma_{1}^{s}(\theta_{0},\phi_{0},v_{0})=\left\{({\hat{y}}_{1},w_{1},\theta_{0},\phi_{0},v_{0})|\;w_{1}=\lambda_{-}(\theta_{0})\left({\hat{y}}_{1}-\frac{b(\theta_{0},\phi_{0})}{p_{+}(\theta_{0})}\right),\;{\hat{y}}_{1}\leq 0\right\},$$

recall (4.5), with λ_−_ defined in (4.6). $\gamma_{1}^{s}$ therefore intersects the *w*_1_-axis in

5.2 $$\gamma_{1}^{s}\cap \{{\hat{y}}_{1}=0\}:\;w_{1}=w_{1*}\equiv -\lambda_{-}(\theta_{0})\frac{b(\theta_{0},\phi_{0})}{p_{+}(\theta_{0})}<0,$$

and divides the negative *w*_1_-axis into (i) initial conditions that lift off directly (*w*_10_>*w*_1*_, dotted cyan in figure 5) and (ii) initial conditions that undergo IWC before returning to $\hat{y}=0$ (*w*_10_<*w*_1*_, purple in figure 5). (A canard phenomenon occurs around *w*_10_=*w*_1*_ for 0<*ϵ*≪1, where the solution follows a saddle-type slow manifold for an extended period of time.) In theorem 3.2, we consider *w*_10_<*w*_1*_. The remainder of the proof of theorem 3.2 on IWC in the indeterminate case then follows the proof of theorem 3.1.

## 6. Discussion

The quantity *e*(*δ*,*θ*_0_) in theorems 3.1 and 3.2 relates the initial horizontal velocity *v*_0_ of the rod to the resulting vertical velocity at the end of IWC. It is like a ‘horizontal coefficient of restitution’. The leading order expression of *e*(*δ*,*θ*_0_) in (3.4) for *δ*≫1 is independent of *μ*, in general. Using the expressions for *q*_±_ and *p*_±_ in (2.6), together with (4.31), we find for large *δ* that

6.1 $$e(\delta ,\theta_{0})=\frac{\alpha }{2(1+\alpha )}\sin(2\theta_{0})\delta^{-2}(1+O(\delta^{-2}\;\ln\;\delta^{-1})),\;\theta_{0}\in (\theta_{1},\theta_{2}).$$

The limit δ→∞ is not uniform in *θ*∈(*θ*_1_,*θ*_2_).

The expression for *δ*≪1 is more complicated and *does* depend upon *μ*, in general. Using (2.6) and (4.32), for *δ*=0, we have

6.2 $$e(0,\theta_{0})=\sqrt{\frac{\left(1+\alpha \;\cos^{2}\;\theta_{0}-\mu \alpha \;\sin\;\theta_{0}\;\cos\;\theta_{0}\right)}{\left(\alpha \;\sin\;\theta_{0}\;\cos\;\theta_{0}-\mu (1+\alpha \;\sin^{2}\;\theta_{0})\right)}\frac{\alpha \;\sin\;\theta_{0}\;\cos\;\theta_{0}}{\left(1+\alpha \;\sin^{2}\;\theta_{0}\right)}}.$$

**Figure 6.**
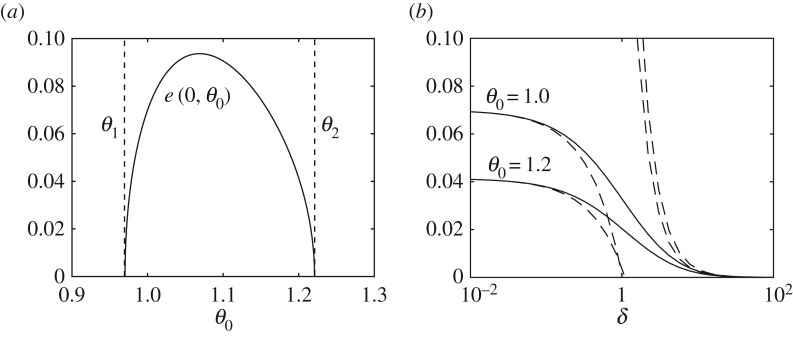
(*a*) Graph of *e*(0,*θ*_0_) from (6.2), where *θ*_1,2_ are given by (2.14). (*b*) Graph of *e*(*δ*,*θ*_0_) for *θ*_0_=1 and *θ*_0_=1.2, where the dashed lines correspond to the approximations obtained from (3.4) and (3.5). For both figures, *α*=3 and *μ*=1.4.

We plot *e*(0,*θ*_0_) in figure 6*a* for *α*=3 and *μ*=1.4. Figure 6*b* shows the graph of *e*(*δ*,1) and *e*(*δ*,1.2) along with the approximations (dashed lines) in (3.4) and (3.5).

In the inconsistent case, described by theorem 3.1, the initial conditions (3.1) are very similar to those assumed by [24]. Interestingly, by applying the approach in §4b backwards in time, it follows that the backward flow of (3.1) for *b*<0 (dashed lines in figure 4, illustrating the *κ*_1_ dynamics) follows *γ*^s^, the stable manifold of *C* for *ϵ*=0, as ϵ→0. Hence, by (4.3)_*ϵ*=0_, the horizontal velocity *v* (and therefore also the energy) increases unboundedly. This ‘backward blowup’ occurs on the fast time-scale *τ*. As a consequence, it is impossible to set up the conditions (3.1) in an experiment without using some form of controller (as was done in [15] for the two-link manipulator system).

**Figure 7.**
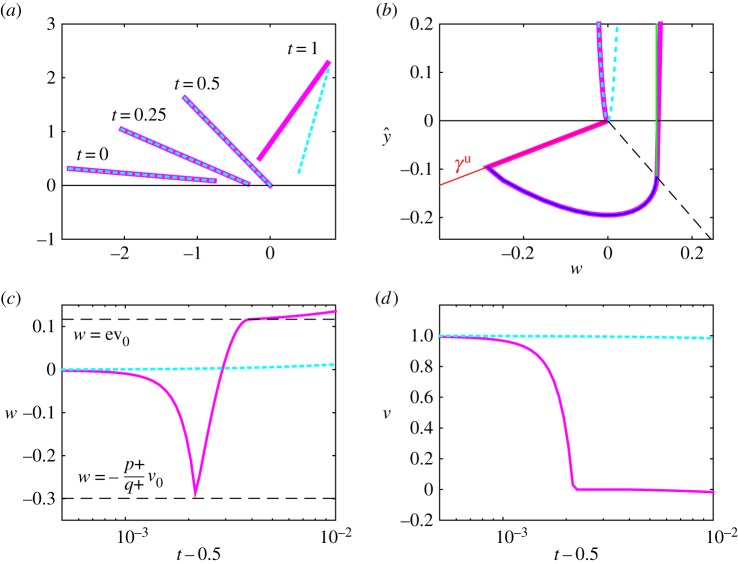
(*a*) Dynamics of the Painlevé rod described by the Filippov system (2.5) for *μ*=*α*=3, *δ*=1 and *ϵ*=10^−3^ in the indeterminate case. The purple and cyan rods are separated at *t*=0 by a distance of 10^−3^. At around *t*=0.5, impact with the compliant surface occurs. The purple rod experiences IWC, whereas the cyan rod lifts off directly. (*b*) Projection onto the $\left(w,\hat{y}\right)$-plane. The blue sticking orbit and the green lift-off orbit from figure 3*b* are also shown. The numerical and theoretical results are indistinguishable. (*c*,*d*) *w* and *v* as functions of time near *t*=0.5 for both rods.

The indeterminate case, described by theorem 3.2, is characterized by an extreme exponential splitting in phase space, due to the stable manifold of *C*_1_ in the *κ*_1_ system. For example, the cyan orbit in figure 5 lifts off directly with w=O(ϵ). But on the other side of the stable manifold, the purple orbit undergoes IWC and then lifts off with w=O(1). The initial conditions in theorem 3.2 correspond to orbits that are almost grazing ($\dot{y}=w=O(\epsilon ),\;\ddot{y}=\dot{w}=b>0$) the compliant surface at *y*=0. In figure 7, we illustrate this further by computing the full Filippov system (2.5)_*ϵ*=10^−3^_ for two rods (purple and cyan as in figure 5) initially distant by an amount of 10^−3^ above the compliant surface (*y*≈0.1, see also *t*=0 in figure 7*a*). We set *μ*=*α*=3, *δ*=1. Figure 7*a* shows the configuration of the rods at different times *t*=0, *t*=0.25, *t*=0.5 and *t*=1. Up until *t*=0.5, the two rods are indistinguishable. At *t*=0.5, grazing ($\dot{y}=w\approx -10^{-3}$) with the compliant surface *y*=0 occurs where *θ*≈0.9463, *ϕ*≈1.6654, and *v*≈1.00 (so *b*≈1.2500 and *p*_+_≈−2.243). The purple rod then undergoes IWC, occurring on the fast time-scale *τ*, and therefore subsequently lifts off from *y*=0 with w=O(1). In comparison, the cyan rod lifts off with *w*≈10^−3^. At *t*=1, the two rods are clearly separated. Figure 7*b* shows the projection of the numerical solution in figure 7*a* onto the $\left(w,\hat{y}\right)$-plane, together with the theoretical predictions of figure 3*b*. Note that the numerical and analytical solutions are indistinguishable, in both the sticking and lift-off regimes. The cyan orbit lifts off directly. The purple orbit, being on the other side of the stable manifold of *C*_1_, follows the unstable manifold (*γ*_*u*_, shown in red) until sticking occurs. Then when ${\hat{F}}_{N}=0$ at $\hat{y}+\delta \hat{w}=0$ (dashed line), lift-off occurs almost vertically in the $\left(w,\hat{y}\right)$-plane. Figure 7*c*,*d* shows the vertical velocity *w* and horizontal velocity *v*, respectively, for both orbits over the same time interval as figure 7*b*; note the sharp transition for the purple orbit around *t*=0.5, as it undergoes IWC. In figure 7*c*, we include two dashed lines *w*=*ev*_0_ and *w*=−(*p*_+_/*q*_+_)*v*_0_, corresponding to our analytical results (3.3) and (4.9), which also hold for the indeterminate case (from theorem 3.2), in excellent agreement with the numerical results.

## 7. Conclusion

We have considered the problem of a rigid body, subject to a unilateral constraint, in the presence of Coulomb friction. Our approach was to regularize the problem by assuming a compliance with stiffness and damping at the point of contact. This leads to a slow–fast system, where the small parameter *ϵ* is the inverse of the square root of the stiffness.

Like other authors, we found that the fast time-scale dynamics is unstable. Dupont & Yamajako [21] established conditions in which these dynamics can be stabilized. By contrast, McClamroch [28] established under what conditions the unstable fast time-scale dynamics could be controlled by the slow time-scale dynamics. Other authors have used the initial scaling (4.1), together with the scaling *κ*_1_ to numerically compute stability boundaries [21,28] or phase plane diagrams [6].

The main achievement of this paper is to rigorously derive these, and other, results that have eluded others in simpler settings. For example, the work of Zhao *et al.* [24] assumes no damping in the compliance and uses formal methods to provide estimates of the times spent in the three phases of IWC. They suggest that their analysis can ‘⋯ roughly explain why the Painlevé paradox can result in [IWC]’. By contrast, we assumed that the compliance has *both* stiffness and damping, analysed the problem rigorously, derived exact and asymptotic expressions for many important quantities in the problem and showed *exactly* how and why the Painlevé paradox can result in IWC. There are no existing results comparable to (3.3)–(3.5) for any value of *δ*.

Our results are presented for arbitrary values of the compliance damping, and we are able to give explicit asymptotic expressions in the limiting cases of small and large damping, all for a large class of rigid bodies, including the case of the classical Painlevé example in figure 1.

Given a general class of rigid body and a general class of normal reaction, we have been able to derive an explicit connection between the initial horizontal velocity of the body and its lift-off vertical velocity, for arbitrary values of the compliance damping, as a function of the initial orientation of the body.

## Footnotes

1 We prefer to avoid describing this phase of the motion as *sliding* because we will be using ideas from piecewise smooth systems [4], where sliding has exactly the opposite meaning.

2 Compare with fig. 2 of Génot & Brogliato [17], where the authors plot the *unscaled* angular velocity *ωϕ* versus *θ*, for the case *g*=9.8 ms^−2^, *l*=1 m.

3 More accurately, the chart $\bar{y}=-1$ corresponds to

$$r=r_{1}\sqrt{1+\epsilon_{1}^{2}+w_{1}^{2}},\;\bar{\hat{y}}=-\frac{1}{\sqrt{1+\epsilon_{1}^{2}+w_{1}^{2}}},\;\bar{w}=\frac{w_{1}}{\sqrt{1+\epsilon_{1}^{2}+w_{1}^{2}}}\;\mathrm{and}\;\bar{\epsilon }=\frac{\epsilon_{1}}{\sqrt{1+\epsilon_{1}^{2}+w_{1}^{2}}},$$

with ${\bar{\hat{y}}}^{2}+{\bar{w}}^{2}+{\bar{\epsilon }}^{2}=1.$ See [34] for further details on directional and scaling charts.

4 Note that (4.13) is not a *blowup* transformation in the sense of Krupa & Szmolyan [35], where geometric blowup is applied in conjunction with desingularization to study loss of hyperbolicity in slow–fast systems. We will not desingularize the vector-field here.
